# A review on Deep Learning approaches for low-dose Computed Tomography restoration

**DOI:** 10.1007/s40747-021-00405-x

**Published:** 2021-05-30

**Authors:** K. A. Saneera Hemantha Kulathilake, Nor Aniza Abdullah, Aznul Qalid Md Sabri, Khin Wee Lai

**Affiliations:** 1 Department of Computer System and Technology, Faculty of Computer Science and Information Technology, Universiti Malaya, 50603 Kuala Lumpur, Malaysia; 2 Department of Artificial Intelligence, Faculty of Computer Science and Information Technology, Universiti Malaya, 50603 Kuala Lumpur, Malaysia; 3 Department of Biomedical Engineering, Faculty of Engineering, Universiti Malaya, 50603 Kuala Lumpur, Malaysia

**Keywords:** Deep Learning, Generative adversarial networks, Optimization, Medical datasets, Structure preservation, Denoising

## Abstract

Computed Tomography (CT) is a widely use medical image modality in clinical medicine, because it produces excellent visualizations of fine structural details of the human body. In clinical procedures, it is desirable to acquire CT scans by minimizing the X-ray flux to prevent patients from being exposed to high radiation. However, these Low-Dose CT (LDCT) scanning protocols compromise the signal-to-noise ratio of the CT images because of noise and artifacts over the image space. Thus, various restoration methods have been published over the past 3 decades to produce high-quality CT images from these LDCT images. More recently, as opposed to conventional LDCT restoration methods, Deep Learning (DL)-based LDCT restoration approaches have been rather common due to their characteristics of being data-driven, high-performance, and fast execution. Thus, this study aims to elaborate on the role of DL techniques in LDCT restoration and critically review the applications of DL-based approaches for LDCT restoration. To achieve this aim, different aspects of DL-based LDCT restoration applications were analyzed. These include DL architectures, performance gains, functional requirements, and the diversity of objective functions. The outcome of the study highlights the existing limitations and future directions for DL-based LDCT restoration. To the best of our knowledge, there have been no previous reviews, which specifically address this topic.

## Introduction

Computed Tomography (CT) is one of the reliable and non-invasive medical image modalities that help to detect pathological abnormalities in the human body such as tumors, vascular diseases, lung nodules, internal injuries, and bone fractures. In addition to the diagnostic support, CT is also useful in guiding various clinical procedures, including interventions, radiation therapies, and surgeries [38]. However, repeated CT scans may reveal that the patient may be exposed to radiation enormously. Overexpose to the radiation would cause the development of metabolic abnormalities, radiation-induced cancer, and other genetic disorders that fall the patients’ quality of life rapidly [75]. Therefore, low-dose CT (LDCT) scanning protocols have been proposed to minimize patients’ exposure to radiation while maintaining adequate diagnostic accuracy.

Usually, to obtain the LDCT images, the X-ray flux is being reduced deliberately during the clinical procedures [55, 57]. The reduction of X-ray flux will degrade the Signal-to-Noise Ratio (SNR) of the X-ray signals and result in low-contrast CT images with noise and artifacts. These visual degradation cause blurring of the edges and losses of contrast within the organs and textures [11]. As a result, the reliability of both the clinical diagnostic procedures and automated analysis tasks such as segmentation, feature extraction, and classification of these LDCT images are deteriorated [38]. However, to overcome these visual degradations and improve the clinical usability of the LDCT images, there are various denoising algorithms have been proposed over the past 5 decades. Overall, those algorithms can be divided into three categories, such as sinogram domain filtering, iterative reconstruction, and image domain processing [52].

In general, the CT restoration methods map the LDCT images back to their’ Normal-Dose CT (NDCT) representations. However, the limited access to projection data in the sinogram domain and high computation cost in the iterative reconstruction domain make the LDCT restoration restricted. Compared to this, image domain processing follows the image post-processing approach and does not rely on projection data. However, the mage domain-based algorithm degrades its performance by estimating the noise distribution according to a specific noise model as part of the noise reduction process. Recently, Deep Learning (DL) has become state-of-the-art in medical imaging. It plays a vital role in solving various problems, including image denoising, super-resolution, detection, and recognition [64, 88]. The rapid growth of hardware technology, the rising need for high-performance processing, data-driven execution, and the ability to crack the previously resolvable problems have dramatically accelerated the resurgence of DL in medical imaging [43]. Hence, much attention has recently been paid to proposing new LDCT restoration algorithms using various DL techniques.

Our survey of relevant works has revealed that very few reviews have recently been published to discuss the conventional general CT denoising methods [11, 38]. With the emergence of DL techniques, most of these conventional denoising algorithms discussed in those reviews are technically obsolete concerning the several LDCT restoration aspects such as accuracy of noise reduction, the ability of lesion discrimination, and the preservation of the fine structure and texture details. Also, to the best of our knowledge, there is no previous study done to date for reviewing the role of DL on LDCT restoration and how those restoration aspects impact in LDCT restoration. Thus, this study reviews DL-based LDCT restoration articles published on the web of science indexed journals starting from the first article published in 2017.

The main contributions of this review are fourfold: (1) analyzing the potentials of DL techniques and architectures used in LDCT restoration; (2) highlighting the specific contributions of DL-based LDCT restoration applications concerning the model performance, structure preservation, and lesion discrimination; (3) reviewing the diversity of objective functions for making different LDCT restoration decisions; (4) discussing the limitations and future research directions to emphasize the existing knowledge gaps.

The rest of the article is organized as follows. The section “Overview of LDCT restoration” provides a brief overview of the degradations in LDCT images. The section “DL architectures” elaborates on different DL techniques and their architectures that were used in LDCT restoration applications. The section “Datasets and methods to deal with data related issues” presents the commonly used datasets and the methods used to overcome some shortcomings of these datasets for DL-based LDCT reconstruction. The section “Diversity of loss functions” discusses the diversity of loss functions in this domain of research. The section “Functional aspects” presents the performance and results of different functional requirements of the proposed applications. The section “Methods for fine-tuning the performance” describes the most commonly used methods for fine-tuning proposed LDCT restoration models. Finally, the section “Future research directions” presents the limitations and future research directions.

## Overview of LDCT restoration

### LDCT imaging

**Fig. 1:**
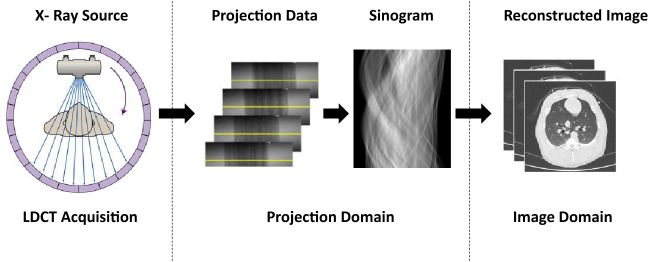
A diagram of the CT imaging

CT scan is an X-ray procedure that creates 2D or 3D cross-sectional images with the help of computer processing. CT scans are more detailed than the conventional X-ray and can reveal shape, dimensions, density, and internal defects of the various anatomies [11]. Figure 1 depicts a diagram of the CT imaging. Accordingly, the CT scanner uses a motorized X-ray source that shoots narrow beams of X-rays as it rotates around the patient. There are special digital X-ray detectors located directly opposite the X-ray source. As the X-rays pass through the patient, they are picked up by the detectors and transmitted to a computer. These transmitted projection data are further processed through radon and inverse radon transform. Also, the back-projection algorithm is applied during this process to reconstruct as CT images. Finally, the reconstructed image slices can either be displayed individually in 2D form or stacked together to generate a 3D image. Analyzing and correcting the CT image quality after reconstruction are a mandatory post-processing task. This is mainly caused by the reduced reconstruction quality that is affected by the reduction of X-ray tube current which is done to prevent patients from adverse radiation exposure.

### Degradations in LDCT

**Fig. 2:**
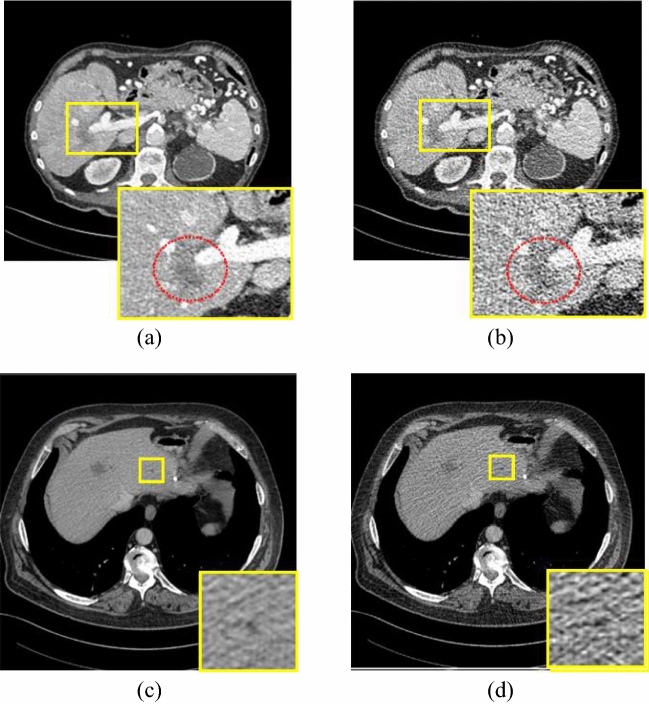
Visuals of CT degradations. **a**, **b** Normal dose and quantum noise corrupted abdomen CT image (The metastasis in of liver lesion marked in a red circle is unclear.) [77]; **c**, **d** normal dose and quantum noise corrupted abdomen CT images with streak artifacts [73]

In general, CT images are degraded by quantum noise and various artifacts during LDCT acquisition. Among them, the quantum noise is embedded in LDCT due to the X-ray photon starvation during the image acquisition [11]. Disconnecting the edges, smoothing the target subtle structures and forming the low-contrast visuals due to lack of X-ray photons are the visual degradations of quantum noise. Figure 2b depicts the consequences of quantum noise in the real abdomen quarter dose CT image for further clarifications. Physically, the quantum noise presents non-uniform distribution over the image space. As a result, validation and learning of the LDCT restoration algorithms become challenging due to the difficulty of distinguishing the actual noise content in CT images [45]. Usually, the quantum noise is approximated by Poisson distribution during experimenting [11]. In addition to that, there are some applications in which the noise distribution of CT images is estimated by considering the Mixed Poisson Gaussian distribution (MPGD) [38]. In MPGD, both the electronic noise and quantum noise components will be modeled using the Gaussian and Poisson distributions, respectively [10].

Apart from the noise, the LDCT images are degraded by blurring [13, 60, 73] and streaking artifacts [28, 34, 50, 71, 75, 81, 91]. Lack of X-ray photons during the CT scanning and patient motion cause blurring. Furthermore, it makes some obstructions in the detection of subtle structures, for instance, liver lesions [73]. The streaking artifact presents as several dark streaking bands placed between two solid objects in the LDCT image (Fig. 2d). Usually, it occurs along the long axis of a high attenuation object. The X-ray beam hardening is the root cause of the streaking artifact.

### A brief overview of conventional methods

Many LDCT restoration methods have been proposed over the past few decades and all of those can be categorized into three groups, namely sinogram domain filtering, iterative reconstruction, and image domain restoration [52]. In general, the sinogram domain filtering-based restoration methods directly work out on the raw projection data that formed before the back-projection. Hence, the restoration algorithms are efficient and can compute the noise statistics accurately. Structural adaptive filtering [37, 70], bilateral filtering [47], and penalized likelihood method [68] are the popular sinogram domain filtering methods. However, these projection data are vender specific and cannot be publicly accessed. Also, the LDCT images restored through sinogram domain filtering suffer from edge blurring and low contrast.

Iterative reconstruction depends on the image’s prior information and performs noise reduction by iterating between the sinogram and image domain. Non-local means [5], total variation [89], dictionary learning [74], and low-rank approximation [2] are some of the priors used within the iterative reconstruction-based restoration category. Even though this LDCT restoration category outputs exciting CT enhancement results, the high computation cost and content loss are the reported drawbacks of iterative reconstruction-based CT restoration.

Compared to the first two restoration categories, image domain-based restoration is considered as a post-processing method. Thus, the restoration algorithms are directly applied to reconstructed images instead of raw data. Conventional image denoising methods such as non-local means [84, 90], total variation [32], Block Matching Three Dimension (BM3D) [26], and statistics-based algorithms [19] are well-known algorithms grouped under this category. Even though the image domain restoration methods are flexible enough to be implemented, the inability to compute the noise statistics due to its non-uniformity will deprive the accuracy of the proposed CT restoration applications. Furthermore, it obscures the structural information of the CT images enormously. Hence, the current LDCT restoration methods and their limitations have paved the direction for proposing novel LDCT restoration methods.

### Emergence of Machine Learning

Machine Learning (ML) is a branch of Artificial Intelligence that facilitates the application to automatically learn and improve through experience rather than using the user-defined programs. ML achieves this automatic learning via a technique called feature learning. The objective of feature learning is to assist the ML application in automatically finding the representations required for solving the target ML problem. It refers to the determination of the optimal model parameter set *θ* that contains a set of candidate solutions (weights) *w* and bias *β* (i.e., *θ* = *(w, β)*) [45]. Generally, this goal is achieved through an objective function that is specifically developed for the target ML model.

Initially, shallow neural networks, such as functional link artificial neural network models, were proposed for medical image restoration. Relying on prior domain knowledge of the problem to be solved is a special feature of those models. However, determining this prior knowledge was somewhat challenging when applying these models for CT restoration. The main reason for that is there was no specific way to determine the noise distribution across the image domain. Thus, there was no any LDCT restoration application has reported based on the shallow neural networks. Later, the DL has become the state-of-the-art of ML in parallel to the improvement of GPU technology and the growing demand for high-performance processing. As a result of this progressive technology development, LDCT restoration has also recently undergone a revolutionary change.

DL is known as the representation-learning method. It lets the computer automatically find the representations from the raw data required for classification and detection. Thus, the DL model consists of multiple levels of feature representations (multiple hidden layers except for the input and output layers) starting with raw input to a more abstract higher level [41]. Thus, this high-level feature capturing of DL models demonstrates its ability to learn the uncertain noise distributions over the LDCT images throughout the data-driven learning. Besides, the data-driven learning method can adapt to any noise type effectively [83]. Hence, it improves the overall performance of LDCT restoration and possesses a novel advantage over other LDCT restoration methods [6, 46].

## DL architectures

**Fig. 3:**
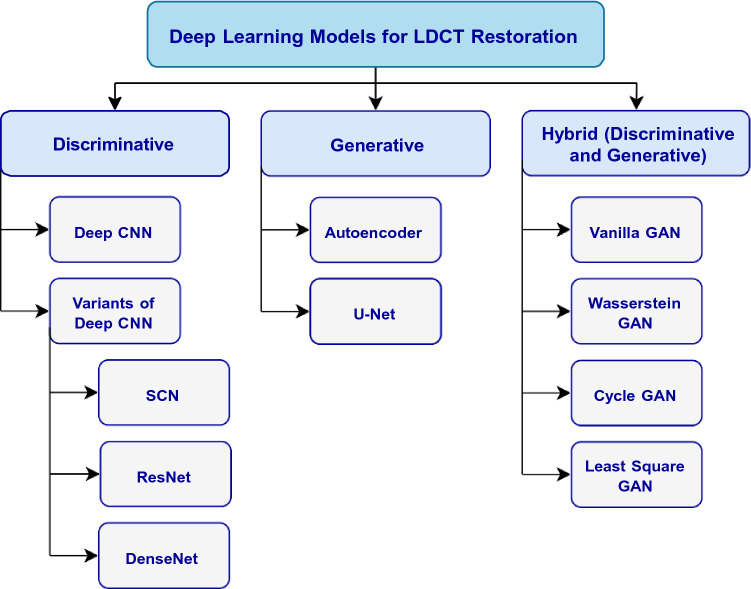
Classification of DL methods used for LDCT restoration

Depending on the network model adopted, DL-based LDCT restoration methods surveyed in this study can be divided into three sub-categories, namely discriminative, generative, and hybrid (generative and discriminative) [61]. Figure 3 depicts the classification of various DL models used for LDCT restoration.

### Discriminative models

**Fig. 4:**
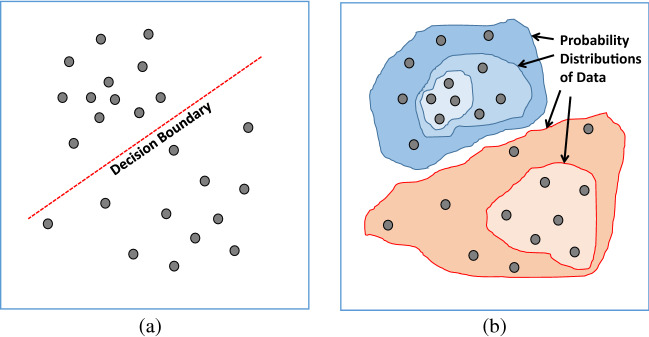
Functional difference of DL techniques: **a** model based on the discriminative approach; **b** model based on the generative approach

The network models based on the discriminative approach represent bottom–up execution to separate learned data based on a decision boundary [61]. Figure 4a depicts the functional aspect of a typical discriminative model. Also, the training strategy of the discriminative approach follows the supervised learning that relies on labeled or annotated data to determine the learning function or prediction model that maps input data to output. Furthermore, in this review, Convolutional Neural Networks (CNN) and their variant have been found as the discriminative models used in LDCT restoration. Table 1 summarizes the discriminative model-based LDCT restoration applications for further information.

**Table 1:** Analysis of discriminative model-based LDCT restoration applications

References	Model features						Strength	Weaknesses
	Network design	Input	Model depth	Shortcut	BN	Objective function		
CNN200, Chen et al. [4]	CNN	33 × 33 sparse patches	3	No	No	L3	Simple architecture	Over smoothing [3]
Du et al. [13]	SCN	100 × 100 patches from LDCT	Stacked competitive blocks	No	No	L3	Multi-scale processing with great structure preservation	Competitive blocks increase the training parameters due to redundant feature maps
AAPM-Net, Kang et al. [36]	Deep CNN	55 × 55 × 15 patches from contourlet transformed image	24	6 bypass connections, concatenation connection	Yes	L3	Better performs in contrast to the model-based iterative reconstruction	Over smoothing. Consuming considerable computation time due to the wavelet transform
Yang et al. [78]	ResNet	128 × 128 cropped images and noise patches	12	Bypass connections	Yes	L3	Both 2D and 3D network models are independent of the input size	The model over fits during the testing
Wu et al. [73]	Cascaded CNN	LDCT images	Cascaded CNN	Cascade connections	Yes	L3	Cascade NN suppresses the blocky and steak artifacts	False lesion artifact
WaveResNet, Kang et al. [34]	Wavelet-based ResNet	55 × 55 × 15 patches from contourlet transformed image	24	Bypass connections Convolution blocks, Concatenation connection	Yes	L3	High network convergence	resistance to generalizability
Wang et al. [71]	RLNet [86] + Wavelet + ConvNet	38 × 38 × 22	13	No	Yes	L3	Outperforming the statistical reconstruction methods Reduction of the high-frequency noise artifacts	Lack of generalizability (Tested only for two clinical tasks)
GRCNN, Gou et al. [23]	CNN [86]	LDCT images	*	No	Yes	L1, L16	Adapting to various noise types and preserve subtle structures	Finding the optimal model depth is laborious
DRL-E-MP, Gholizadeh-Ansari et al. [20]	Dilated CNN based on RLNet [86]	40 × 40 sized overlapping patches	8 dilated convolution layers	Concatenation connections	Yes	L3, L8	Dilation convolution allows capturing more contextual details using fewer layers	The perceptual loss was determined using VGG16 which trained using natural images
DP-ResNet, Yin et al. [81]	Projection domain (SD-Net) and image domain (ID-Net)-based ResNets	Overlapping 3D blocks of noisy cone-beam projections for SD-Net	For SD-Net = 9, for ID-Net = 11	For SD-Net = Bypass connections, for ID-Net = Bypass connections and a contracting path connection	SD-Net = Yes	L3, L4	Incorporating the projection domain and image domain has enhanced the restoration results	False lesion artifact
DCRN, Ming et al. [50]	Densely connected ResNet	LDCT images	12	Skip connections via the concatenation layer	Yes	L3	Preserving the structural details	Dense-connections increased the depth of the network, and it leads to a long training time
TLR-CNN, Zhong et al. [91]	ResNet with Transfer Learning	40 × 40 sized patches	15	No	Yes	L3	Generalizability and performance enhancement via transfer learning	Streaking artifacts and noise is remaining in the processed images
Shiri et al. [60]	ResNet with dilated convolution	512 × 512 voxels	20	Bypass connections	Yes	L3	Providing clear visualizations of lesions in the lung when reducing the radiation dose index by up to 89%	Failure to recover the correct structure details of the lesion
TS-RCNN, Huang et al. [30]	Two-stage ResNet with stationary wavelet transform	512 × 512 × 4	9	Bypass connections	Yes	L3, L8	Preservation of the structural details	Weak texture details
CT-ReCNN Jiang et al. [33]	Multi-scale parallel RerNet with dilated convolution	100 × 100 sized patches	Shallow channel: 5, deep channel: 13	Bypass connections	Yes	L2 (among residual images)	Preserved multi-scale detailed features texture details of lung images	Complex architecture

#### CNN

**Fig. 5:**
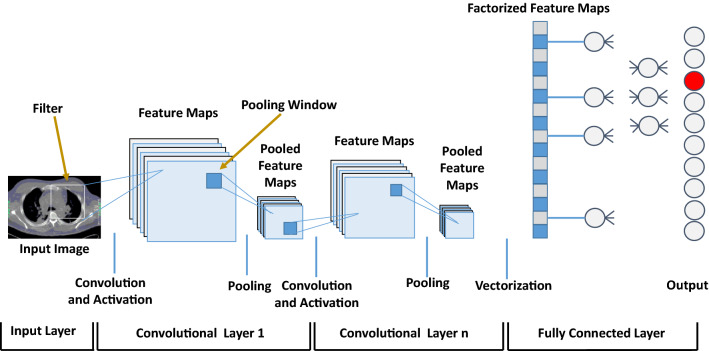
Generic architecture of the CNN model

Due to the recent advancement in high-performance computing and hardware resources, CNN-based denoising applications have popular in medical imaging [65]. It takes 2D or 3D images as input and better utilize the structural details greatly for feature extraction and processing. As shown in Fig. 5, CNN is organized based on three consecutive implementation components, namely the convolutional layer, the pooling layer, and the fully connected layer [59]. The convolution layers apply the mathematical operation called “convolution” over the image to generate the feature maps. These generated feature maps consist of local features such as edges, object boundaries, and various texture patterns that are spatially distributed within LDCT images. To achieve this, the convolutional layer uses multiple filters which are deployed as stacked layers, in the same layer. Thus, CNN helps to enhance the input noisy images by focusing on the local image details. This spatially adaptive enhancement reduces the noise embedded in the processed images. The main function of the pooling layer is to effectively reduce the dimensions of the generated feature maps. These are kept robust to the geometry and position of the detected features within the processed image. Finally, the output of CNN is generated by fully connected layers. This is achieved by integrating all the feature maps or responses formed by the previous processing steps [29].

In LDCT restoration, CNN attempts to learn a mapping function between LDCT and NDCT images by optimizing the objective function on a training dataset [18]. Thus, the convolution layers with multiple filters and pooling layers are common in CNN-based LDCT restoration models. Furthermore, in LDCT restoration, the densely connected layers found in the generic CNN model are replaced with an output layer followed by a suitable activation function. Chen et al. [4] have proposed a simple and effective CNN-based LDCT restoration method that works on LDCT images (CNN200). It has performed patch-by-patch-based mapping between LDCT and NDCT images during the restoration.

#### Variants of CNN

Improving visual performance and gaining optimal network training are the ever-growing requirements in LDCT restoration. However, it has been revealed that the generic CNN model has a lack of architectural support to achieve these requirements. As a solution for this, the variants of CNN architectures have been published. The following sections briefly explain the significant aspects of those CNN architectures for further clarifications.

**Fig. 6:**
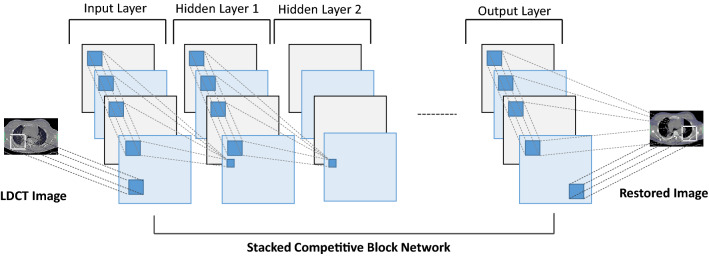
Generic architecture of the SCN model

**Stacked Competitive Network (SCN):** The SCN consists of a multi-stacked layered architecture that is formed by a set of successive competitive blocks [13]. This feature emphasizes the main difference between SCN and generic CNN. Furthermore, as shown in Fig. 6, each competitive block in SCN has introduced multi-scale processing. The objective of a single competitive block is to enhance the local structural details within the competitive block with a certain sparsity. Thus, it has increased the width of the CNN and enabled to extract of more low-level details in the LDCT images.

Multi-scale conventional filters that operate within competing blocks can capture information about the multi-scale structural features and textures of the same LDCT image region. Furthermore, a combination function is implemented in each block to minimize the redundant feature capturing and reduce the computational load. The objective function of the proposed network was designed to minimize the competitive mapping of each layer of the proposed SCN network. Furthermore, it consists of a regularization term to control over-fitting. Reconstructed CT images through this proposed SCN model visualize sharp edges and better distinguish low-contrast structures effectively.

**Fig. 7:**
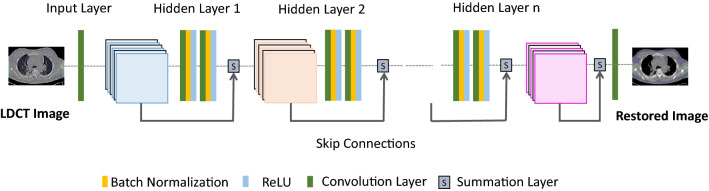
Generic architecture of the ResNet model

**Fig. 8:**
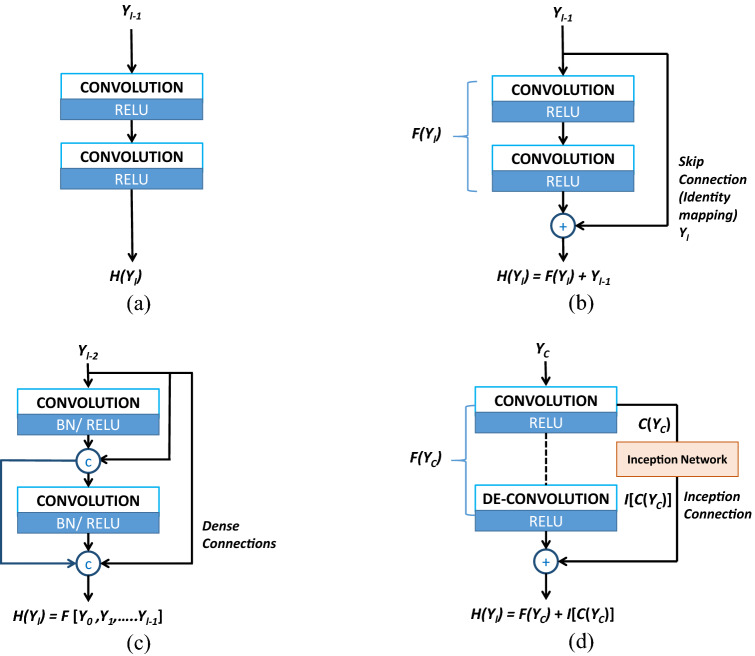
Different shortcut connections. **a** CNN with sequential convolution layers, **b** ResNet with convolution block and skip connection. *Y*_*l*_—input from the *z* residual unit, *Y*_*l*+*1*_—output from *l* + *1* unit, *F*(*Y*)—residual mapping of the stacked convolutional layer. **c** DenseNet with dense connections. DenseNet concatenates the output passed from previous layers, **d** inception ResNet connection, and *Y*_*c*_, *C*, *I*, *F* represent the input, convolution, inception filtering, and network operations, respectively

**Residual Network (ResNet):** Stacking more layers in the CNN model is one of the basic techniques for improving the performance of the CNN model. However, increasing the depth of the network will always not influence CNN positively due to the issue called gradient diffusion [20, 50]. Also, gradient diffusion might result in failures in network training. As a solution for this issue, He et al. [27] have proposed the multi-branch network called ResNet. Figure 7 depicts the generic architecture of the ResNet for further clarification. The most notable aspects in the ResNet architecture are the skip connections and residue estimation strategy in which are not common in generic CNNs. Skip connections found in ResNet models transfer the extracted features from the previous layers to the subsequent layers to preserve the structural details. Figure 8a and b depicts this architectural difference between the generic CNN and ResNet with skip connection for further clarifications. The 2D-ResNet proposed by Yang et al. [78] have followed this basic ResNet architecture, and later, they enhanced this network to its 3D version to preserve the spatial co-relation of tissues and organs. Apart from that, the two-stage ResNet (DP-ResNet) published in [81] has implemented two ResNets that performed the LDCT restoration in both the projection domain and image domain. Processing the sinogram data in the first stage of this application enables it to enormously suppress the noise in low-dose projection data. Later, processing the already restored projection data in the image domain has reduced the remaining residues and streaking artifacts greatly.

This study revealed that some of the LDCT restoration applications reported in [20, 23, 71, 73] followed the same ResNet model published by Zhang et al. [86]. Accordingly, a cascaded ResNet-based LDCT restoration model published by Wu et al. [73] has the strength to restore the noise patterns that would rarely encounter in the training datasets via iterative cascaded learning. In addition to that, Gou et al. [23] (GRCNN) has proposed a gradient regularization-based objective function to the model suggested in [86]. Hence, the proposed GRCNN has gained the training effectiveness and ability to preserve the sharpness of features of the processed LDCT images. In addition to these applications, the ResNet published by Gholizadeh-Ansari et al. [20] (DRL-E-MP) has some unique features compared to other applications that followed the model in Zhang et al. [86]. Those are edge-detection-based image restoration and the application of dilated convolution operations. In addition to that, the study done by Shiri et al. [60] has also used dilation convolution for the ResNet proposed to enhance the COVID-19 CT data. Moreover, the multi-scale parallel CNN model proposed by Jiang et al. [33] has also used the dilated convolution to denoise the lung images. This model not only reduces the noise but also preserves the detailed features of the low-dose lung CT with texture details. The implementation of two parallel networks, three different sized convolution kernels, and residual connections are the significant architectural aspects that support gaining this visual performance. The ability to increase the receptive field of dilation convolution impact these studies positively to preserve more contextual details in the LDCT images.

Except for pure ResNet-based LDCT restoration applications, some studies have been published that combine ResNet with wavelets. The prime objective of such an integration is to restore the texture details and eliminate the noise-induced artifacts in ultra-LDCT images. Among them, the AAPM-Net model in [36] has been developed based on the high-frequency channels obtained after contourlet transformation on the LDCT images. Furthermore, in this application, the lower frequency wavelet coefficients were then integrated with the denoised frequency bands to reduce unnecessary load on the model. Later, the Wave-ResNet has been published as an extension to the AAPM-Net [34]. Estimating the residuals at each sub-band by the ResNet and implementation of concatenation later in the network are the specific features in Wave-ResNet in contrast to AAPM-Net. Apart from that, the two-stage denoising model (TS- RCNN2) in [30] has been trained using the stationary wavelet transformed LDCT and averaged-NDCT images. The two ResNets in this application have performed texture preservation and structure enhancement, respectively.

Contrary to the above-mentioned ResNets, the TLR-CNN published in [91] was free from bypass connections. Instead of that, it has fine-tuned the network via a two-stage transfer learning strategy in which the first stage uses the natural images with blind Gaussian noise, and the second stage uses the LDCT images.

**Fig. 9:**
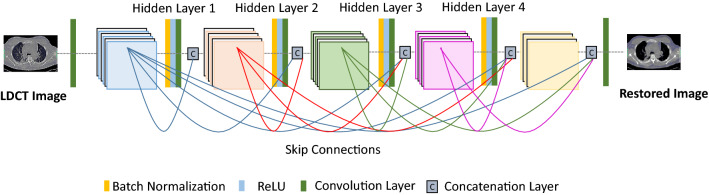
Generic architecture of the DenseNet model

**Dense Network (DenseNet)**: Similar to ResNets, DenseNets are also another way that can use to increase the depth of the network [29]. DenseNet simplifies the connectivity pattern between the input and output layers, so that it can minimize the gradient diffusion issue of the CNNs. In contrast to the ResNet that skips signal from one layer to the next through summation, DenseNet surges information exchange among the layers in the neural network via a simple connectivity model layers of the same feature map size (as shown in Fig. 9). Thus, each layer receives inputs from all preceding layers and sends on its feature maps to all successive layers. Moreover, it boosts the network’s feature learning capability and the reusability of feature maps. Because of that, the subsequent layers of the network can use the full feature maps of all initial layers. Therefore, this aspect in DenseNet will tremendously help to reduce the information loss during the training. Figure 8c depicts the functional point of view of a typical dense connection in a network. Contrary to the DenseNet in [29], Ming et al. [50] have proposed a DenseNet for LDCT restoration by reducing the connectivity pattern to gain computational efficiency in each block while training the network.

**VGG19:** VGG19 is a pre-trained CNN published by Simonyan, Zisserman [63], which consists of 16 convolutional layers followed by the three fully connected layers. The output of the last convolutional layer of the VGG19 is the feature map of the input image. In LDCT restoration, the VGG network is used for computing the perceptual loss [12, 58, 78, 79].

As a summary of the facts mentioned in Table 1, it can be stated that the discriminative models preserve the fine structures in the restored CT images and reduce the streaking artifacts greatly. However, the structures are over-smoothed due to the MSE-based objective function. Also, the ResNet-based studies have degraded the results due to the lack of generalizability.

### Generative models

DL models categorized under the generative approach determine the probabilistic distribution of data. Compared to the discriminative approach, the generative approach shows the top–down execution. Furthermore, it follows the unsupervised learning strategy for feature learning (Un-supervised learning performs learning on the input data itself rather than using annotated data.). Figure 4b depicts the functional aspect of a typical generative model for further clarifications. In this study, the autoencoder and U-net models were identified as the widely used generative models for LDCT restoration.

#### Autoencoder

**Fig. 10:**
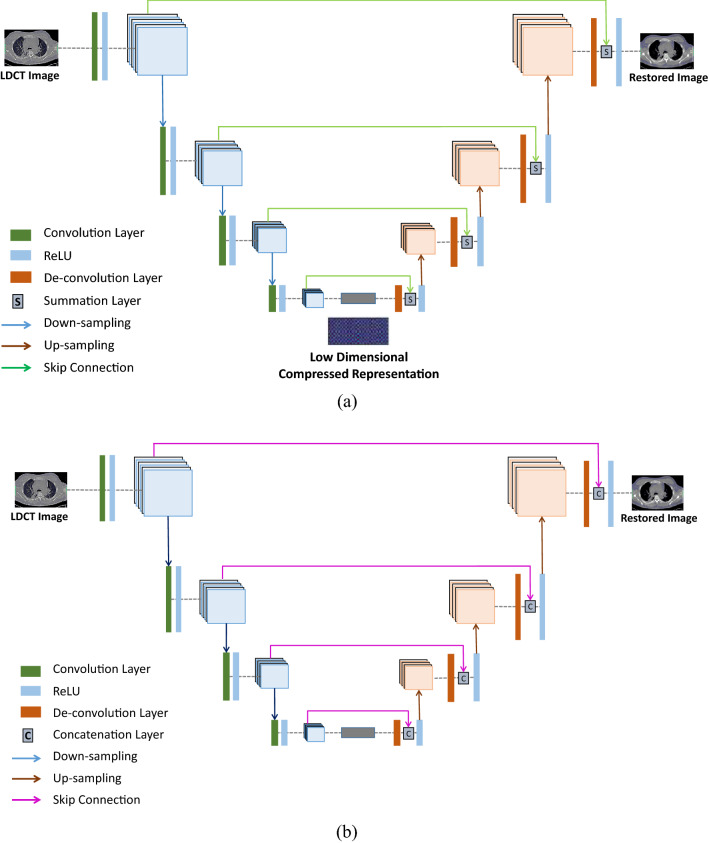
Generic architecture of generative models used for LDCT restoration: **a** autoencoder; **b** U-Net

Autoencoder learns how to compress and encode input data and then learns how to reconstruct the output data back from the compressed encoded representation. Hence, it gets the output representations that are much similar to the original data. As shown in Fig. 10a, the architecture of the autoencoder consists of two components, namely encoder, and decoder. Out of these two components, the encoder is made up of a set of fully connected or convolutional layers. In LDCT restoration, the encoder performs the feature extraction from noisy LDCT images and transforms the image data into a low-dimensional compressed representation called a bottleneck. After that, the decoder up-samples the low-dimensional representation to reconstruct the denoised image using fully connected layers or convolutional layers. In training, autoencoders regenerate the input data itself using the backpropagation algorithm [61]. Like ResNet, the autoencoder network has also connected corresponding encoder and decoder layers with skip connections. As a result, the network depth has increased and minimized the gradient diffusion that happens during the training.

Recently, Mao et al. [49] have published an autoencoder (RED-Net) that can restore natural images degraded by different noise levels. Based on that, later, Chen et al. [3] have published an RED-CNN model by combining autoencoder with CNN for LDCT restoration. Unlike the reference model in [49], this RED-CNN model has removed the Rectified Linear Unit (ReLU) layers before the summation with residuals to ignore the positivity constraint on learned residuals. In addition to that, Liu, Zhang [45] proposed an LDCT restoration method based on the Stacked Sparse Denoising Autoencoder (SSDA) model. On the contrary to the autoencoders, SSDA adds a sparsity component based on the Kulback–Leibler divergence to the learning model. Thus, it supports content preservation optimally. Moreover, the proposed SSDA model did not contain any down-sampling layer and was made up of using a shallow network structure. Different from all the CNN-based DL models published for LDCT restoration, Fan et al. [16] have proposed a stacked autoencoder model based on the quadratic neurons (Q-AE). The replacement of the conventional neurons with quadratic neurons in this Q-AE has motivated to represent complex data, and it has positively influenced to enhance the robustness of LDCT restoration. Also, the quadratic operation has boosted the processing power of the individual neurons. Except for the application of quadratic neurons, the proposed network model of Q-AE is fundamentally similar to the RED-CNN. Also, interested readers can find more information about quadratic neurons from [14, 15, 17]. Overall, it is significant to state that all the cited autoencoder applications in this section have used MSE (L3) as the loss function. Furthermore, Table 2 summarizes the autoencoder-based generative DL applications for further analysis.

**Table 2:** Analysis of generative model-based LDCT restoration applications

References	Model features				Strength	Weaknesses
	Network design	Input	Model depth	Shortcut		
RED-CNN, Chen et al. [3]	Residual EnDec	LDCT images	5 Convolution and 5 De-convolution layers	Long skip connections	Enhancing the low-contrast regions	Texture loss and blurring due to the usage of MSE-based objective function False lesion issue
Liu, Zhang [45]	Stacked sparse denoising Autoencoder	8 × 8 sized patches	3 stacked sparse denoising autoencoder (6 hidden layers)	No	Preserving the texture details in which decays during the down-sampling	The proposed model still distorted some subtle structures
Q-AE, Fan et al. [16]	Quadratic Autoencoder	64 × 64 sized patches	5 quadratic convolution and 5 quadratic deconvolution layers	Bypass connections	Low computational cost due to the lower number of training parameters	Determining the depth of the network is laborious

#### U-Net

Ronneberger et al. [56] have proposed the U-net model, which consists of symmetric architecture constructed by a contracting path and expanding path. As shown in Fig. 10b, the contraction path comprises convolution operations and down-sampling layers, while the expanding path consists of up-sampling layers. Hence, the contracting and expanding paths resemble the encoder and decoder layers, respectively. U-net consists of long skip connections to transfer the feature details from the encoder layers to the corresponding decoder layers. Unlike the residual skip connections, these transferred features finally concatenate at the corresponding decoding layer. Different from residual connections, the concatenation type skip connections in U-net allow transferring of more feature information forward, and it is a significant performance aspect in U-net architecture [44]. Furthermore, it has been observed that almost all of the U-net-based LDCT restoration applications reviewed in this study have been published by integrating U-net with the Generative Adversarial Network (GAN) s [6, 45]. However, after publishing the Pix-to-Pix GAN by Isola et al. [31], there were several LDCT restoration applications published based on it. The main reason for that is the generator of the Pix-to-Pix GAN followed the U-net architecture, and it accepts an image as the input instead of the noise distribution in the latent space [75, 79]. The deeper U-net published in [79] permits to retain of the small details of the processed LDCT images.

### Hybrid models

**Fig. 11:**
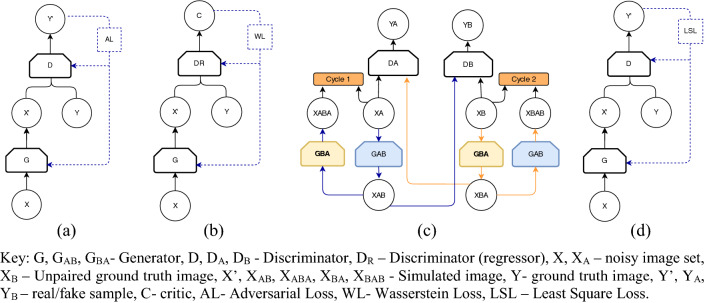
Variant of GAN architectures: **a** Vanilla GAN, **b** WGAN, **c** Cycle-GAN, and **d** LS-GAN

The hybrid learning approach combines both the generative and discriminative network models to construct the learning model. After introducing GAN by Goodfellow et al. [22], this hybrid learning model has become popular in LDCT restoration. The GAN consists of two CNN models, which are defined as the generator and the discriminator [22]. In medical image denoising, the generator synthesizes the samples from learning the distribution of low-dose medical images. The discriminator receives both the normal dose images and the synthetic images produced by the generator and aims to distinguish them apart [8]. This basic structure of GAN is known as vanilla GAN. Moreover, GAN is flexible to implement different generator models based on various CNN architectures, such as the encoder–decoder [58, 67], U-Net [6, 53, 75, 79], and ResNet [12, 28, 46]. Also, the discriminator mostly acts as a binary classifier to distinguish the synthetic and NDCT images apart. Depending on the adversarial learning method and the objective function used, several variants of GAN architectures have been published. Our review of literature has revealed Wasserstein GAN, cycle GAN, and least-square GAN as the variants of GAN which are broadly used in LDCT restoration. Figure 11 depicts the network model of each of these GANS and Table 3 summarizes the important features of the GAN-based LDCT restoration applications.

**Table 3:** Analysis of GAN-based LDCT restoration applications

Publication	GAN Model	Input	Generator design			Discriminator design	Losses in objective function	Strength (s)	Weakness (s)
			Depth	Shortcut	BN				
Wolterink et al. [72]	Vanilla Gan	Voxels of size 65 × 65 × 19	CNN with 7 convolution layers	No	Yes	CNN with 11 convolution layers	L2, L5	Texture preservation	Quality degradation due to the usage of Wasserstein distance [79] Ring artifact
WGAN-VGG, Yang et al. [77]	W-GAN	Patches of size 64 × 64	CNN with 8 convolution layers	No	No	CNN with 6 convolution layers [63]	L6, L8	VGG loss prevents severe distortions	False lesion issue [6]. Generating some distorted results [83]
SAGAN, Yi, Babyn [79]	Vanilla Gan	Patches of size 256 × 256	UNet256 [31] including 21 convolution layers and 2 deconvolution layers)	9 bypass connections in 9 residual blocks	Yes	Patch-GAN structure of [31] (Patch size is 70 × 70)	L5, L9	Ensures the sharpness	The sensitivity of the sharpness metric is low in some low-contrast regions. False lesion issue [6]
CPCE- 2D/3D, Shan et al. [58]	Vanilla Gan	Patches of size 64 × 64	3D conveying path-based convolutional encoder-decoder network. 4 encoder and 4 deconvolution layers	Three conveying paths between the corresponding encoder and decoder layers	No	CNN with 6 convolutional layers	L5, L8	2D-to-3D transfer learning method is efficient and stable [72]	Loss of texture and tissue details [21]
SMGAN, You et al. [83]	W-GAN	Voxels of size 80 × 80 × 11	3D CNN with 8 convolution layers	No	No	CNN with 6 convolutional layers	L4, L6, L7	It successfully preserves the critical features to be diagnosed	Edge blurring and structural dissimilarities appeared in some cases
Kang et al. [35]	Cycle GAN	Patches of size 56 × 56	CNN with 20 convolution layers	6 bypass connections in 6 convolution blocks	Yes	Patch-GAN structure of [31] (Patch size is 70 × 70)	L5, L10, L11	Resistance to mode collapse and robust to cardiac motion and contrast change	Lack of texture preservation due to the low stability of the DL model [75]. Altering the local image details [80]
m-WGAN, Hu et al. [28]	W-GAN	Patches of size 64 × 64	ResNet with 16 Residual blocks (38 Convolution layers)	16 Bypass connection in 16 residual blocks	Yes	CNN with 8 convolution layers	L3, L6	Minimizing the over-smoothing	The noise and artifacts remain in the restored images
Park et al. [53]	Vanilla GAN	Patches of size 128 × 128	U-net like deep convolutional framelet	3 bypass connection, contracting paths connect low-frequency bands	Yes	Patch-GAN structure with 4 convolution layers	L5, L17	Preserving the detailed information during the down-sampling, and fidelity term protects the subtle details	Gaussian noise components cannot be successfully removed by the proposed method [53]
VAGAN, Du et al. [12]	Vanilla GAN	Patches of size 64 × 64	Residual Encoder-Decoder Network with 14 convolution blocks	14 Bypass connections in 14 convolution blocks	No	Attentive discriminator with 8 convolution layers	L5, L8, L12, L13, L14	The network pays special attention to the noisy regions and background structures [6]	VAGAN performance towards PSNR is weak
CycleGAN-BM3D, Tang et al. [67]	Cycle GAN	Patches of size 128 × 128	Encoder-decoder network with 18 convolution layers	6 Bypass connections in residual blocks	No	CNN with 5 convolution layer	L5, L10, L15	BM3D-based prior information prevents the generation of incorrect details	Cycle GAN alters the local image details [80]
HFSGAN, Yang et al. [75]	Least Square GAN	Images with size 512 × 512	Two nested U-Nets	7 Bypass connections between corresponding encoder and decoder layers	Yes	Patch-GAN structure of [31] with 2 convolution layers and 3 inception modules	L4, L17, L18	The inception-based discriminator boosts the accuracy of the generator Preserving more texture details	Running two parallel U-net models has increased the model complexity
Ma et al. [46]	Least Square GAN	Patches of size 55 × 55	ResNet with 14 convolution layers	5 bypass connections in the 5 residual blocks	No	CNN with 6 convolution layers	L4, L7, L18	Minimizing the vanishing gradient and allows content learning with the SSIM	Lack of performance in pixel-level similarity assessment
Chi et al. [6]	Least Square GAN	Patches of size 256 × 256	Modified U-net with inception residual blocks (4 convolution and 4 deconvolution layers)	4 bypass connections to concatenate inception residual blocks with deconvolution layers	Yes	Multi-layer CNN	L3 (between noise and content), L5, L8	Preserving the structural details and eliminates false lesions	Noise is still retained in the restored CT images
SACNN, Li et al. [42]	W-GAN	Stacked 3 patches of size 64 × 64	CNN with 8 convolution layer and 3 3D self-attention blocks	3D self-attention blocks	No	CNN with 6 convolution layers	L6, L8	Improving the denoising performance of both GAN and CNN-based networks	Low PSNR and SSIM for some cases due to not using the pixel-wise loss function
Gu, Ye [24]	Cycle- GAN	Patches of size 128 × 128	U-net generator with 10 convolution layers and AdaIn layers	By pass connections to connect encoder layers with corresponding decoder layers	No	Patch-GAN structure with 6 convolution layers	L10, L11, L18	One generator, Stable training with less number of parameters	Structure preservation need to be improved further
DRWGAN-PF Yin et al. [82]	W-GAN	Patches of size 128 × 128	CNN with 8 convolution layers. 2nd and 6th layer consists of dilated convolution	3 bypass connections between the 3 convolution blocks	Yes	CNN with 6 convolution layers	L2, L6, L8*	Trained on unpaired data	Perceptual loss is determined using natural images (VGG-19 net)

*Multi-perceptual loss—average perceptual loss of 5 levels in VGG-19 net

#### Vanilla GAN

Vanilla GAN represents the simplest GAN model as depicted in Fig. 11a. Wolterink et al. [72] have first applied the GAN for resolving the limitation of voxel-wise regression in LDCT noise reduction. Later, Yi, Babyn [79] have proposed a GAN model by conditioning it with sharpness loss to enhance the edges and boundaries of the structural details, which are pathologically significant. Also, Shan et al. [58] have proposed a conveying path-based GAN model that can integrate the 3D spatial details via the adjacent 2D LDCT slices. In this application, first, the 2D LDCT restoration model has been proposed and the strong correlation of those 2D slices was used as a transfer learning to train the 3D model. LDCT restoration application published in [53] is significant, because it has addressed the issue of lacking the paired medical image data (low-dose images and identical ground truth images) for training the GAN models. The fidelity embedded GAN model proposed by Park et al. [53] for LDCT reconstruction has computed the Kullback–Leibler divergence and L2 loss to generate the denoised CT images by training the GAN through unpaired CT images. The application of visual attention for image restoration is still novel in the CT domain. Du et al. [12] were the first team who have applied the attention network to overcome the over-smoothing caused by MSE loss function in current DL-based CT restoration models. The generated attention map of this study was used as prior knowledge about noise distribution over the input image and the implemented visual attention block sustained in the proposed restoration model not only to preserve the fine structures (lesions and other subtle structures) with perceptual similarity but also to explicitly assess the local consistency of the recovered regions [6].

#### Wasserstein GAN

In general, minimizing the generator of the vanilla GAN is equivalent to minimizing the Jason–Shannon divergence between noisy and ground truth data distribution. However, it has been revealed that minimizing the Jason–Shannon divergence has led to a vanishing gradient on the generator network and obstruct updating as the training continues. To overcome this, Arjovsky et al. [1] proposed the Wasserstein distance between noisy and ground truth data, which has been formulated based on the geodesic distance of the degraded and ground truth data distributions. Later, with the modification added by [25], Wasserstein distance was used with GAN and has called WGAN (Fig. 11b). Furthermore, in this study, several WGAN-based LDCT restoration models have been analyzed. Those were performed various additional functional aspects such as enhancement of perceptual similarity [77], preservation of structural details [83], and reduction of low-dose artifacts in dental CT images [28].

In general, the CNN-based restoration methods are inherently less efficient in modeling various structural information in CT images due to the non-uniformity of noise distribution and the mixture of texture and the geometric shapes of CT images. Also, the fixed-size filtering in current CNN-based restoration methods unavoidably keeps some irrelevant pixels for the current response, especially for the regions with complex structures and the edges. Besides, training algorithms may have problems coordinating dependencies across different layers, making weight learning inefficient as a result. Li et al. [42] have proven the strength of solving the mentioned issues through a self-attention model by establishing interactions between the local outputs and all other pixels within one layer to guide the convolutional filtering. The proposed method consists of two attention networks named plane attention and depth attention for dealing with long-range dependencies within the CT slice and among the CT slices, respectively. Furthermore, contrary to the computing VGG-based [63] perceptual loss in [77], the proposed model consists of a self-supervised learning scheme for assessing perceptual similarity. The restored CT images contain sharp edges, fine texture details, and no waxy artifacts. Apart from that, Yin et al. [82] have proposed a W-GAN model based on unpaired data to denoise Lung CT images. Noise reduction and texture preservation of this proposed GAN model were boosted by the residual connections and the multi-perceptual loss computed based on the VGG-19 network.

#### Cycle GAN

Cycle-GAN(C-GAN) was proposed by Zhu et al. [92] and has gained extensive attention in image enhancement. It tends to focus on the spatial features of one collection of images and decides on how to map those learned elements to another image collection without the need for trained pair of examples (degraded and corresponding terrain real images). Different from other GAN models, C-GAN architecture consists of two generators and two discriminators, as shown in Fig. 11c. Unlike conventional GAN models, adversarial learning is not useful for C-GAN. The main reasons for that are, first, there was nothing to constraint the generator to synthesize the final content irrespective of the ground truth image, and second, whatever the image synthesized by the generator was well enough to fool the discriminator best. Thus, the objective of C-GAN would be extended to ensure that the restored image still looks like the ground truth in some way. As a consequence, the cycle consistency loss has been added to the two generators in C-GAN. Thus, the first generator restores the image according to the way it feels necessary and the second generator learns alongside how to restore that synthesized image to its original representation. In this learning process, both generators update their weight based on the difference between the unpaired ground truth image and the synthesized images. This way of learning ensures that the main generator does not disregard its input completely, and using the second generator allows for flexibility in that restoration process.

Literature has revealed the application of C-GAN-based LDCT restoration models in the studies done by Kang et al. [35] and Tang et al. [67] (CycleGAN-BM3D). Accordingly, those studies tend to restore the LDCT images by learning the distributions of the unpaired collection of NDCT images. Among them, the C-GAN model proposed by Tang et al. [67] has applied a BM3D-based image before minimizing the risk of synthesizing the false details by the first generator. Furthermore, contrary to other GAN models, the C-GAN can minimize the mode collapse due to the usage of inversion paths. Unlike the conventional C-GAN model with two generators, the recent C-GAN model proposed by Gu, Ye [24] has used U-net based single generator for LDCT noise reduction. Using the Adaptive Instance Normalization (AdaIN) layers to execute the low-dose to high-dose image translation by switching to the generator model is the significant architectural improvement in this proposed model.

#### Least square GAN (LS-GAN)

Mao et al. [48] have proposed LS-GAN as an extension of vanilla GAN by changing the loss function for the discriminator to least-square loss instead of binary cross-entropy. Thus, except for the loss function, the network architecture of the LS-GAN is exactly as same as the vanilla GAN as shown in Fig. 11d. The binary cross-entropy loss function is unable to evade the vanishing gradient issue in GAN due to its failure to generate a strong signal to best update the model. To overcome this issue, the least-square loss has been used as the loss function, and it will penalize the synthesized images according to their distance from the decision boundary. Hence, the least-square loss objective function gains the ability to generate a strong gradient signal for the generated samples located far from the decision boundary. As a result of the strong gradient, those samples distal to the decision boundary are moved closer to the decision boundary and form enhanced images as an output. Moreover, our study of literature has clearly emphasized several LS-GAN-based LDCT restoration applications [6, 46, 75].

Among these studies, Yang et al. [75] have implemented two U-net-based generators for their application named HFSGAN. The objective of the first generator of this study is to process the high-frequency bands of LDCT to improve the generators’ sensitivity for high-frequency details. Then, the second generator of the HFSGAN synthesizes the restored CT images by combining the priory processed high-frequency bands and low-frequency bands of the LDCT images. Also, different from other GAN-based applications, HFSGAN has proposed a multi-scale discriminator with an inception module [66], to extract the multi-scale features of LDCT images. Apart from that, the LS-GAN suggested by Chi et al. [6] has used inception residual blocks in the generator network to prevent transferring noise in each convolution layer to the deconvolution layer via shortcut connection. Moreover, Fig. 8d shows an architectural diagram of how to connect the inception block to the bypass connection for further explanation. Apart from that, to increase the performance, this application has a discriminator with a multi-level joint architecture.

Almost all of the GAN model presented in Table 3 consists of multi-objective functions. As a result, those individual learning models can enhance the different features during restoration. Furthermore, it can be observed that most W-GAN-based DL models have not been used the batch normalization during generator design. Also, Patch-GAN and Cycle-GAN models have used U-net or Encoder–Decoder type GAN models for generator design. Overall, all of the GAN models were capable to restore the fine details of the LDCT images and preserve the texture and artifacts.

## Datasets and methods to deal with data-related issues

### Techniques for boosting the training samples

DL relies heavily on large training datasets to reaching high learning accuracy [45]. Table 4 summarizes the standard datasets found in reviewed LDCT restoration applications. However, the amount of data associated with these datasets are not sufficient to gain high performance in LDCT restoration. Therefore, various solutions have been implemented to increase the availability of CT data for effectively training and validation of DL models.

**Table 4:** Common datasets used in the reviewed literature

ID	Dataset	Anatomy	Remarks	Related studies
*Public datasets*				
D01	NBIA/NCIA dataset [54]	Many organs including, Chest	The National Biomedical Imaging Archive consists of 7,015 total NDCT images of 256 × 256 size. URL: https://imaging.nci.nih.gov/nbia-search-cover/	[3, 4, 6, 13, 20, 45, 50, 79, 91]
D02	AAPM-Mayo	Abdomen	Mayo clinic AAPM Low- Dose CT Grand Challenge dataset consists of 2378 full and quarter dose CT images from 10 patients of 512 × 512 size. URL: https://www.aapm.org/GrandChallenge/LowDoseCT/	[3, 6, 12, 13, 16, 23, 30, 34, 36, 42, 46, 58, 71, 73, 75, 77, 78, 81, 83, 91]
D03	Piglet dataset [79]	Whole-body	Images were obtained under four dose levels and each dose level consists of 850 images of 512 × 512 size	[20, 75, 79]
D04	Data Science Bowl 2017	Lung	The dataset consists of over a thousand high-resolution LDCT images of high-risk lung cancer patients. https://www.kaggle.com/c/data-science-bowl-2017/data	[79]
D05	3D-IRCADb	Different organs	1375 clinical NDCT images of 10 patients. URL: https://www.ircad.fr/research/3dircadb/	[30]
D06	Luna-16	Lung	888 clinical NDCT scans are available with annotations. URL: https://luna16.grand-challenge.org/Data/	[82]
*Private datasets*				
D06	Cardiac CT	Cardiac	Cardiac CT scans of 28 patients	[72]
D07	MGH dataset [76]	Abdomen, chest, and head	Massachusetts General Hospital (MGH) dataset consists of 40 cadaver scans obtained under four dose levels	[58]
D08	Cardiac CT	Cardiac	Two sets of 50 CT scans of mitral valve prolapse and coronary artery disease patients	[35]
D09	Dental CT	Dental CT	CT images were reconstructed using sinogram data in axial, sagittal, and coronal planes	[28]
D10	Liver simulated dataset	Liver and portal vein	2480 NDCT images of liver and portal vein of 62 patients	[53]
D11	Brain clinical dataset	Brain	200 brain CT images of two different dose levels (100 from each.)	[53]
D12	Piglet dataset	Whole-body	360 data were scanned under four dose levels	[67]
D13	COVID-19	Lung	1141 volumetric chest CT exams were obtained from 9 medical centers. Among them, 312 data were marked as PCR-positive	[60]

Paired CT datasets of normal dose and low dose are essential for the training and validation of DL models. The repetitive scanning of patients is the only possible way to extract NDCT data in clinical procedures. However, this is not permitted in clinical practice, because prolonged exposure to radiation can adversely affect patients' quality of life. Also, CT sinogram data are vendor-specific and are not permitted to be extracted from third parties. However, to overcome this challenge, several applications have suggested techniques to use unpaired training data and noise priors for training the DL models [35, 53, 67]. In addition to that, the non-reference metrics are suitable for quantitative evaluations. The reason for that is those matrices are free from measuring the similarity between LDCT and NDCT images during the performance evaluation [7].

Also, recent DL applications have used simple geometric transformations-based data augmentation techniques [3, 4, 45] and image patching methods as the techniques for boosting the amount of training data in the limited number of medical datasets. In data augmentation, the use of scaling as a data augmentation technique may change the size of the original image, resulting in the risk of losing the CT image in detail [23]. Thus, some studies only focused to apply rotation and flip to increase the number of samples in training datasets [23, 34, 36, 50]. In contrast to data augmentation, patch-based training increases the network convergence [23]. Furthermore, it facilitates to enhance the detection of the perceptual variances in local regions and alternatively increases the number of training samples [3].

### Methods for simulating LDCT

**Fig. 12:**
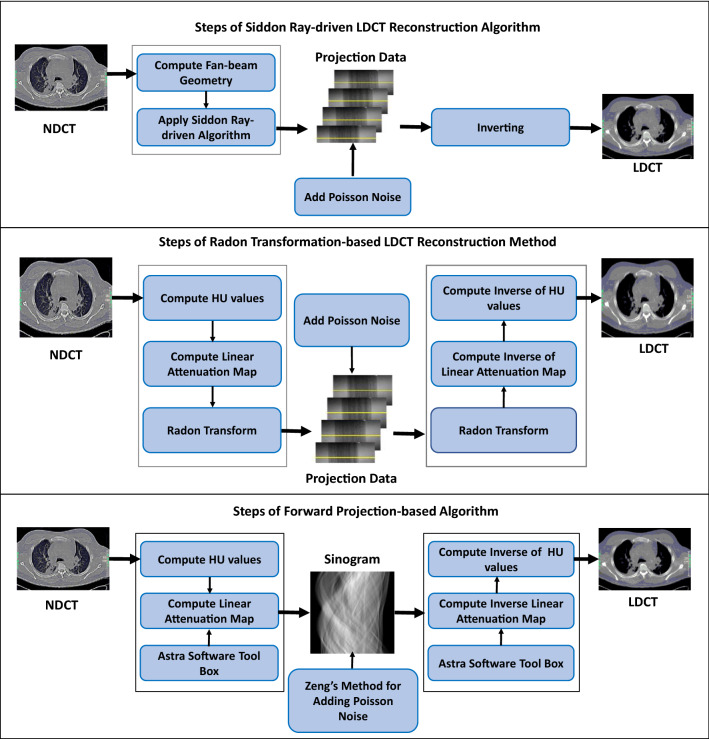
Steps of LDCT reconstruction algorithms

Supervised DL models must have NDCT and its low-dose versions for training and validation. Since it is not practical to get the clinical data as a whole, the reconstruction of LDCT is the acceptable solution for generating the LDCT data. Adding Poisson noise into the sinogram obtained from NDCT is the main function of a typical LDCT reconstruction algorithm, because Poisson noise is the dominant noise type in the LDCT image in the sinogram domain [87]. Depending on the transformation methods used to simulate the sinogram data, there are three main LDCT reconstruction algorithms widely used in LDCT restoration. Those are Siddon ray-driven algorithm [62], radon transformation-based algorithm, and forward projection-based algorithm. Figure 12 depicts the steps of these three LDCT reconstruction methods for further clarifications.

Among these LDCT reconstruction algorithms, the forward projection-based algorithm depends on the external toolbox called Astra [69] and performs well with GPU support. In addition to that, this algorithm follows Zeng’s method [85] to add the Poisson noise into the NDCT sinogram. However, the Siddon ray-driven algorithm and radon transformation-based algorithms simulate the Poisson noise into the low-dose transmission data as a product of simulated low-dose scan incident flux and the exponential of inverse sinogram. The studies [20, 30] have used the radon transform-based algorithm, whereas [91] has used the forward projection-based algorithm for LDCT reconstruction.

## Diversity of loss functions

The objective function in the DL model represents the basic formal specification of the problem to be solved. It consists of two components, namely the regularization term *λ* and the loss function *L*(*θ*). The regularization term of the objective function is used for tolerating the over-fitting of the model. In general, the loss function evaluates how well the data can be modeled in a specific DL model according to the desired enhancement requirements. Hence, an objective function would consist of single or many loss functions. Table 5 lists the loss functions and strength of each of them defined in the articles reviewed in this study.

**Table 5:** Loss functions used in the ML-based literature

Abbreviation	Loss function	Name: description	Strength
L1	${\mathcal{L}}_{\mathrm{PixelDiff}}$	Pixel difference: The pixel value difference between the center pixel of the patch in the ground truth image and the generated pixel value by the network	Preserves the gray content in the generated images
L2	${\mathcal{L}}_{\mathrm{SquiredError}}$	Squired error: Supports to keep the properties of differentiability, symmetry, and convexity. Suffer from blurring. Determines element-wise data fidelity loss in image space	Ensures the pixel-level similarity between generated and real images
L3	${\mathcal{L}}_{L2}$	Average/Mean Squared error: compute the MSE loss between the true and generated images	Ensures the differentiability, symmetry, and convexity between the generated image and the real image
L4	${\mathcal{L}}_{L1}$	L_1_ loss: It computes the mean absolute error of two images	It supports to keep the symmetry, convexity, and reduce blurring. However, it forms blocky artifacts
L5	${\mathcal{L}}_{\mathrm{Adversarial}}$	Adversarial loss: Consisted of generator loss and discriminator loss	Improves the stability of the training process
L6	${\mathcal{L}}_{\mathrm{Wasserstein}}$	Adversarial loss with Wasserstein distance: To determine the GAN loss based on earth mover distance	Leverages the training convergence and improves the stability of the training process
L7	${\mathcal{L}}_{\mathrm{Structure}}$	Structural Loss: The loss which finds the best visual patterns in the images	It discourages blurring and preserves the structure and texture details
L8	${\mathcal{L}}_{\mathrm{Perceptual}}$	Perceptual loss: The loss which determines similarity in the features. It is computed based on the features extracted from the pre-trained networks such as the VGG-19 network [63] or custom network [42]	Structure and texture preservation
L9	${\mathcal{L}}_{\mathrm{Sharp}}$	Sharpness loss: The loss which assesses the sharpness loss of the features represented in the images to be processed	Ensures the sharpness of features
L10	${\mathcal{L}}_{\mathrm{Cyclic}}$	Cyclic loss: The loss which permits one-to-one correspondence between the noisy and denoised image	Enforces the inverse relationship of the cycle-GAN model
L11	${\mathcal{L}}_{\mathrm{Identity}}$	Identity loss: Minimizes the alteration loss of input and generated images of each generator model associated with the cycle GAN	Ensure that the properly generated output images no longer change when used as inputs to the same network
L12	${\mathcal{L}}_{AB}$	Attentive block loss: The loss between the image generated by the attentive block and the prior/ attention knowledge	Create the optimal attention map
L13	${\mathcal{L}}_{\mathrm{MS}}$	Multi-scale loss: The loss which computes further structural and contextual information on different scales	Preserve the structural and contextual information on different scales
L14	${\mathcal{L}}_{A}$	Attention loss: The loss between the features extracted from interior layers of the discriminator and the attention map produced at time step *t* in attentive discriminator	Determine the optimal learning of latent noise (attention feature data) distribution
L15	${\mathcal{L}}_{\mathrm{Prior}}$	BM3D Prior loss: L1 loss between the BM3D prior image and the generated image	Enforces the generator to produce the desired output
L16	${\mathcal{L}}_{\mathrm{Gradient}}$	Gradient loss: The loss which determines the high-level feature-based information	Leverage the training convergence
L17	${\mathcal{L}}_{\mathrm{Details}}$	Detail loss: Restoration loss between the high-frequency sub-bands of the ground truth and the high-frequency sub-bands of the generated images	Preserve the edges
L18	${\mathcal{L}}_{\mathrm{LSL}}$	Least square loss: GAN loss of least-square GAN model	Improves the stability of the training process

MSE is the widely used loss function in many generator and discriminator DL models. However, it has revealed that MSE-based optimization consists of the regression-to-mean problem [75]. Thus, it leads to texture information loss, over-smoothing, and false lesion discrimination [3, 4, 36]. As an alternative for MSE, Least Absolute Error (LAE) is ideal for optimizing the DL models. Even if the LAE is also a mean-based matric, like MSE, experimental results have proven that it can overcome the blurring issues caused by the MSE loss [46]. However, restored images obtained through the LAE-based optimized DL model still degrade due to the blocky artifacts. After the publishing of the image net [9] pre-trained networks, namely VGG-16 and VGG-19 [63], the perceptual loss has been introduced to the DL model optimization to overcome the issues raised by both the MSE and LAE. The perceptual loss computes the feature difference between generated and real CT images. However, experiments on applications that rely solely on perceptual loss have shown that restored images have grid-like artifacts. Therefore, perceptual loss has usually used to optimize the DL models by combining them with MSE [20].

Some studies use the Structural Similarity Index Matrix (SSIM) as a loss function to assure the structure preservation capability of the DL model [46, 83]. It performs better than MSE by providing the highest quantitative values for Peak Signal-to-Noise Ratio (PSNR) in visual assessments [46]. Also, computing the SSIM loss in multi-scale allows capturing additional textual and structural details [12]. Similar to the SSIM, sharpness is also another desired loss function in LDCT restoration studies and determines how the learning process optimally preserves the sharp edges [79]. However, the sensitivity of the proposed sharpness loss function is not up to the expected level for the treatment of blurring in some low contrasting regions. Furthermore, it simulates subtle structures as noise. As a result, the existing sharpness loss function leads to erroneous decisions during lesion discrimination [6].

GAN has also gained attention dramatically in recent developments in LDCT restoration. Conventionally, GAN models use the adversarial loss as its objective function and determine how optimal the min–max game between generator and discriminator. However, the empirical studies have proven that the GAN based on adversarial loss resulted in convergence issues [77]. Thus, inspired by [1] and [25], the Wasserstein distance with the gradient penalty has been introduced as the loss function to overcome the identified convergence issues [35, 42, 77]. Apart from that, the LDCT restoration applications done based on cycle-GAN or least-square GAN have used cycle consistency loss and least-square loss as the loss functions [34, 67, 75].

## Functional aspects

### Noise and artifact suppression

Various DL architectures and performance trade-offs affect the noise and artifact reductions in reviewed studies. In general, noise and artifact reduction gained by various DL models have been quantitatively evaluated by the pixel domain-based metrics, namely PSNR and Mean Structural Similarity Index (MSSIM). Table 6 summarizes these aspects with the average PSNR and MSSIM values reported in the reviewed studies to compare the strengths of the reviewed restoration algorithms.

**Table 6:** Summary of functional aspects of reviewed studies

Article	Test dataset (s)	Model feature for noise and artifact reduction	Quantitative analysis		Aspects of structure preservation			
			MSSIM	PSNR	Border (B) or edges (E)	Organs and structures	Textures	Lesions
*Discriminative models*								
Chen et al. [4]	D01	Deep CNN	0.94 ± 0.02	40.57 ± 1.58	B	Fine structural details	×	×
Du et al. [13]	D01, D02	Competitive blocks in the stacked competitive network	0.95	41.92	×	Fine structural details, inter-costal vein	√	√
Kang et al. [36]	D02	Direction wavelet transform determines the directional artifacts	×	34.8 ± 1.5	B	Liver, blood vessels	√	√
Yang et al. [78]	D02	Optimal model depth and dropouts	0.94 ± 0.002	39.53 ± 0.30	E	Continuous structures, blood vessels	√	×
Wu et al. [73]	D02	Applying the cascading for CNN boosts PSNR	0.75 ± 0.001	42.2 ± 0.05	×	Fine structural details	×	√
Kang et al. [34]	D02	Feed forwards and RNN network structure suppressed the streaking artifacts	0.89	38.54	E	Liver, pelvic bone, blood vessels	√	√
Wang et al. [71]	D02	Statistical iterative reconstruction framework and IRL prior information	×	26.77 ± 12.84	E	All structural details	×	×
Gou et al. [23]	D02	The gradient regularization of the structure details	0.91 ± 0.04	36.62 ± 3.245	E	Soft tissues	×	√
Gholizadeh-Ansari et al. [20]	D01, D03	MSE and perceptual loss combined objective function keep more texture details	0.84	35.57	E	Lung, pulmonary vasculature	√	×
Yin et al. [81]	D01, D02	Sinogram domain network (SD-net), and image domain network (ID-net)	0.95 ± 0.01	41.97 ± 1.49	E	Tissues, blood vessels	√	×
Ming et al. [50]	D01	DenseNet [29]	0.94	33.67	×	Bone grove	×	×
Zhong et al. [91]	D01, D02	Stacked residual blocks	0.96 ± 0.012	43.51 ± 0.95	×	Organs in the thorax	×	×
Shiri et al. [60]	D13	ResNet with dilated convolution	0.97	34.0	×	Lung regions	×	√
Huang et al. [30]	D02, D05	Stationary wavelet transformation and ResNet	0.88	31.0	E	Liver, renal inferior vena cava	√	√
Jiang et al. [33]	D02	Multi-scale parallel ResNet. Dilated convolution	0.94	32.75	×	Detailed structures of Lung	√	×
*Generative models*								
Chen et al. [3]	D01, D02	Shortcut connections have preserved the structure	0.97 ± 0.008	44.42 ± 1.21	B	Adrenal gland, blood vessels	√	√
Liu and Zhang [45]	D01	Stacked sparse denoising autoencoder	×	42.32 ± 0.36	E	Fine structural detail, blood vessels	×	×
Fan et al. [16]	D02	Quadratic autoencoder	0.91 ± 0.05	30.82 ± 2.76	×	Lymph nodes, blood vessels	√	√
*Hybrid models*								
Wolterink et al. [72]	D06	GAN network	×	40.45 ± 4.05	×	×	×	√
Yang et al. [77]	D02	Wasserstein distance and perceptual loss-based objective function	0.78 ± 0.03	24.17 ± 0.07	×	×	√	√
Yi and Babyn [79]	D01, D03, D04	Long skip connections	0.86 ± 0.01	27.51 ± 0.74	E	Fine structural details	×	×
Shan et al. [58]	D02, D07	Conveying path-based encoder and decoder model. Transfer learning is applied from the 2D model to the 3D model	0.90 ± 0.03	29.63 ± 1.68	×	Fine structural details, intrahepatic blood vessels	√	√
You et al. [83]	D02	Structure sensitive objective function	0.78 ± 0.04	26.38 ± 1.01	E	Structural details, blood vessels	√	√
Kang et al. [35]	D08	Cycle GAN	×	×	E	Structural details	×	×
Hu et al. [28]	D09	Deep generator with cascaded ResNet	0.95 ± 0.02	30.79 ± 3.03	E	Trabecular bone, tooth root	×	×
Park et al. [53]	D10	Fidelity term in the objective function	0.89 ± 0.02	34.34 ± 0.92	E	Liver, morphological structures of the tissues, portal vein	×	×
Du et al. [12]	D02	Visual attention network	0.98	42.05	×	Fine structural details	×	√
Tang et al. [67]	D12	Cycle GAN and BM3D prior	0.97 ± 0.04	34.35 ± 0.14	E	Tissue, blood vessels	√	×
Yang et al. [75]	D02, D03	Generator with high-frequency domain-based U-Net and discriminator with inception module	0.94 ± 0.004	32.29 ± 0.99	E	Fine structural details	×	√
Ma et al. [46]	D02	Adopting the least-square loss	0.91	32.70	×	Structural details	√	×
Chi et al. [6]	D02	Generator with inception residual mapping-based U-Net and perceptual multi-level joint discriminator	0.98	44.40	×	Structural details	×	√
Li et al. [42]	D02	Plane and depth attention modules	0.78	22.17	E	Structural details, blood vessels	√	√
Gu and Ye [24]	D01	Switchable generator using AdaIn layers	066	30.87	E	Structural details	×	×
Yin et al. [82]	D06	Multi-perceptual loss and residual connections in the generator network	0.70	30.46	E	Structural details	√	×

×—indicates “not tested” state

Among various DL models that were developed for LDCT restoration, cascaded CNN models leverage the noise and artifact reduction far better than the deep CNN models. The experimental results of the study [73] show that increasing the number of cascades in cascaded CNN reduces the blurring artifacts and remove the streak artifacts around the lesions. The reason for that is, the noise embedded in the NDCT images belongs to both training and validation data get further smoothed by the cascaded network structure [73]. In addition to this, if an LDCT image is transformed into the frequency domain, the noise content of the LDCT image will distribute as the high frequencies in LDCT images. Thus, it can be observed that some studies applied wavelet transformation to LDCT images for estimating and removing these noise-induced frequencies iteratively [30, 36, 71, 75]. After the noise frequency filtering, the residual low-frequency information in the LDCT images can process through the DL model.

### Structure preservation

Developing the adaptive denoising algorithms with excellent structure preservation is a significant function in medical imaging, because it facilitates clinicians to interpret medical images robustly [51]. Also, it improves the accuracy of computer-aided diagnosis methods, such as feature recognition and quantitative analysis. Table 6 summarizes the feasibility of reviewed denoising applications for preserving various clinically significant anatomical structures concerning the validation datasets.

Discriminative model-based DL models have performed quite acceptable improvements in organ and structure preservation. Among them, CNN200 [4] and AAPM-Net [36] have improved the visualization of the boundaries of the organs. Also, AAPM-Net has preserved the textures in the liver area. Hence, it made this application easy to locate the liver lesions and location. However, later studies have empirically proven that both CNN200 and AAPM-Net can produce over-smoothed results with loss of texture information [58]. It had happened due to the regression-to-mean problem caused by the MSE-based loss function used in those applications. The SCN suggested in [13] has better distinguish the textures and enhanced the contrast of inter-costal vein in chest images. Apart from that, the Sobel operator used in the GRCNN model helped to locate the edges and has preserved the soft tissues of organs [23]. Furthermore, the implementation of gradient regularization in the GRCNN model has sharped the preserved edges. Out of the published ResNet-based applications, the RED-CNN has preserved the borders of different tissues [3]. Apart from that, the edge detection layer in DRL-EMP added extra sharpness to the preserved edges [20]. Moreover, the combined objective function of the DRL-EMP has leveraged the preservation of more texture details in the validated images. The DP-ResNet provided acceptable texture preservation via the deep convolution applied in the projection domain and image domain [81]. Hence, this application could be able to preserve the texture, especially in the pelvic bones that are degraded by the artifacts. According to Table 6, all the ResNet-based LDCT restoration applications have contributed to preserving various organs and fine structural details.

The generative model-based DL applications have also proven their capability for preserving the subtle structures while restoring the LDCT images. Consequently, the stacked sparse denoising autoencoder model published in [45] has fully preserved the edges of the pelvis without having any blocky or blurring artifact. Moreover, the RED-CNN [3] and Q-AE [16] models have also successfully preserved the texture information of the processed images.

The contribution of the GANs for structure preservation in LDCT is significant in recent LDCT restoration studies (Table 6). This fact is proven by many of the GAN-based LDCT restoration methods reviewed in this study. The recent GAN-based models have achieved this visual performance through various model design aspects. Some of those significant model design aspects were long skip connections in SAGAN [79], content correspondence in WGAN-VGG [77], the structure sensitive objective function in both SMGAN [83] and [46], content fidelity assessed objective function in [53], the structure-oriented gradient regularization in GRCNN [23], and long-range dependencies maintained by self-attention block in SACNN [42]. However, You et al. [83] have proven that WGAN-VGG [77] suffers from content distortion, even though it can preserve structural details. The content mismatch between the CT images and natural images in the VGG-19 pre-trained network [63] during the calculation of perceptual loss was the main reason for this limitation. Apart from the gradient regularization, the application of edge detection has improved the sharpness of the edges in GRCNN [23]. Besides, CycleGAN-BM3D has proven the ability to prevent the generation of false details in the restored LDCT images via the integration of BM3D prior information [67]. Yang et al. [75] have shown that increasing the receptive field of the network and extraction of multi-scale features have positively affected preserving the texture details. On contrary to this, Li et al. [42] have stated that the perceptual loss computed in the attention network can preserve more texture details in contrast to the VGG-loss-based models. Although the GAN-based LDCT restoration methods have gained a high performance in structure preservation, false lesion issue still affects to degrade the visual quality of the restored LDCT images [6].

### Lesion discrimination

Lesion discrimination is also one of the needful functional requirements in LDCT restoration. It allows clinicians to recognize the various characteristics of the lesion, including the location, shape, border, and density. The improvement of the contrast done by the DL-based restoration models separates the lesion from both the background texture and noise components effectively. Also, the results obtained from qualitative evaluations (visual performance comparisons and blind reader studies) have been used to elaborate on the significance of the identification of lesions in past research studies (Table 6).

Among the discriminative model-based LDCT restoration models, AAPM-NET has first evaluated the detection rate (73%) of focal hepatic lesions of abdomen CT images via a blind reader study [36]. The stacked competitive CNN model in [13] and the cascade CNN model in [73] have also improved the contrast of the lesions in abdomen CT images. Among them, the cascade CNN model [73] has greatly improved the metastasis near the chest regions. In addition to that, the GR-CNN model has improved the shape of the lesion due to the usage of gradient regularization within the CNN model [23]. Also, this study noted that the use of MSE-based loss functions in CNN models has negative consequences for locating the lesion. Also, the WaveResNet [34] enables to locate the lesion due to its ability to preserve the textures. Recently, Shiri et al. [60] have done experiments based on the COVID-19 positive chest CT images. They have proven that the proposed ResNet-based DL algorithm was capable of enhancing the visual clarity of the nodular and wedge shape lesions under ultra-low-dose cases.

The generative model-based LDCT restoration methods have also improved the visual clarity of the lesions. Especially the focal hepatic lesions that appeared in abdomen CT images were enhanced and evaluated in Q-AE [16]. Also, the empirical results have proven the ability to do lesion discrimination by RED-CNN [3]. However, Chi et al. [6] have proved that the lesions enhanced by RED-CNN looked over-smoothed. The main reason for that is the MSE-based objective function used to train the RED-CNN network.

The impact of the GAN for lesion discrimination in LDCT restoration algorithms is outstanding. This statement is proven by the first GAN-based LDCT restoration method proposed by [69], because it has visualized the cardiac artery calcification lesions. Also, the WGAN-VGG [77] and SMGAN [83] models have successfully visualized the metastasis of the liver lesions and cystic lesions in the upper part of the kidney [77]. Moreover, the SMGAN has improved the sharpness of the metastasis in liver lesions due to the structure preservation-based objective function. Apart from that, the validation results of CPCD-3D [58] have proven the visual enhancement of the focal hepatic lesions that appeared in abdomen CT images due to the implementation of 2D-to-3D network-based transfer learning. The attention networks introduced to the GAN models were also supported to enhance the visualization of low attenuation liver lesions. The main reason for that is the efficient noise reduction ability of those networks gained through the attentive blocks [12, 42]. Apart from that, the recent study, HFSGAN [75], has validated the proposed GAN model for the real piglet dataset [79] to show its ability to enhance visualization of the lesions in the real CT images.

In LDCT restoration, generating false lesions is a common issue in ResNet and GAN-based LDCT restoration models [6, 71, 81]. It happens due to the resembling of some noise-induced artifact to view as lesions. In this scenario, the DL model fails to distinguish the difference between the artifact and the real lesion. As a consequence, the diagnosis results might generate false-positive results. WGAN-VGG [77] and SAGAN [79] are two such methods, which suffer from false lesion problem. As a solution, the study published in [6] has proposed inception residual blocks and residual mapping to the U-net based generator to overcome generating unnecessary artifacts. Also, the multi-level joint discriminator introduced in the same study [6] has maintained a constraint to detailed reproduction. As a consequence, it results in better structure preservation excessively. Apart from that, the false lesion issue can generate by the discriminator during the computation of the similarity between the ground truth images and generated images on one scale. This happens due to the tiny noise component distributed over the Ulta-LDCT images. However, the study published in [6] mentioned that simultaneously computing the difference between the output from every down-sampling and corresponding deconvolution layer as a loss of whole U-net-based generator model can also be used to overcome this false lesion issue.

## Methods for fine-tuning the performance

### Shortcut connections

The main function of the shortcut connection (also known as a bypass or skip connection) in the DL model is to pass the output of one layer as input feature maps to the subsequent layers by skipping some layers in the model. Figure 8 depicts those different shortcut connections for visually comparing the architectural variances. Furthermore, Tables 1, 2, and 3 mention the different types of shortcut connections used in the reviewed LCDT-restoration applications. In general, the shortcut connection can preserve more structural information and has a positive effect on improving the visual performance of LDCT images. Furthermore, the skip connections used in the ResNet model help to minimize the gradient vanishing problem thoroughly [36, 81].

### Adaptive learning rates

Learning rate is a critically important hyperparameter that can leverage the optimizer for rapid converging of the DL model. Choosing a too-small value for learning rate may result in a long training process that could get stuck the training process, whereas a too-large value may result in an unstable training process. Thus selecting an optimal value for learning rate is a challenging task. In LDCT restoration applications, the learning rate is associated with the well-known optimizers such as Stochastic Gradient Descent, ADAM [39], and limited memory BFGS [40] algorithm. Many of these LDCT restoration applications are designed to dynamically update the learning rate while training the DL model. These dynamic learning rates reduce the over-fitting and speed up the network convergence [35]. This study has revealed different learning rate scheduling techniques used in LDCT restoration, namely time-based [3, 6, 50, 81], drop-based [16, 20, 35, 78, 91], and exponential-based [58, 72] techniques. Dynamic learning rates reduce the over-fitting and speed up the network convergence [36]. Table 7 summarizes the training and execution efficiency of some of the reviewed studies.

**Table 7:** Summary of training and execution efficiency of reviewed studies

Article	Hardware platform	Handling learning rate	Data augmentation	Tainting dataset size	Batch size	Epochs	Training time (h)	Execution time (s)
*Discriminative models*								
Chen et al. [4]	GPU	Time-based	Rotation (45°), flipping (vertical and horizontal), scaling (2, 0.5)	200 slices (10^6^ patches)	×	×	17	2.05
Kang et al. [36]	NVIDIA GeForce Titan, 6 GB	Time-based	Random flipping and rotate	200 slices (10^6^ patches)	10	50	×	1.6
Yang et al. [78]	NVIDIA GTX 1080, 8 GB	Step-based	×	5080 slices	1	150	×	0.3 (2D model), 0.25 (3D model)
Kang et al. [34]	NVIDIA GeForce GTX 1080 Ti	Time-based	Flipping (vertical and horizontal)	3236 slices	10	×	20	2.05
Wang et al. [71]	Titan X GPU	Step-based	×	22,800 slices for T-net and 433,200 slices for A-net	256	×	8.10 for 2D model, 10.15 for 3D model	×
Yin et al. [81]	NVIDIA GTX 1080, 8 GB	Time-based	×	10^6^ and 10^7^ patches	×	×	4 (10^6^ patches), 10 (10^7^ patches)	0.13 SDnet, 1 for IDnet
Ming et al. [50]	NVIDIA GeForce GTX 1080 Titan	Time-based	Rotation, flipping	100 slices	1	64	6.57	0.002
Zhong et al. [91]	NVIDIA GeForce GTX Titan Xp	Time-based for first 25 epochs and fixed learning rate for next 25 epochs	Rotation, flipping, scaling	5761 slices	128	50	29.5 (30 residual blocks)	0.1
Shiri et al. [60]	NVIDIA GTX 2080 Titan	Adaptive	×	1141 slices	×	10	50	×
Huang et al. [30]	NVIDIA GTX1080 Titan	Time-based	×	1944 slices	4 (stage 1), 16 (stage 2)	55 (stage 1), 30 (stage 2)	4	< 3.68
*Generative models*								
Chen et al. [3]	NVIDIA GTX1080	Time-based	rotation (45°), flipping (vertical and horizontal), scaling (2, 0.5)	10^7^ patches	×	×	30	3.68
Liu, Zhang [45]	NVIDIA GTX 980 Titan	BFGS algorithm	rotation (30°), flipping (vertical and horizontal), scaling (2, 0.5)	300 slices (10^6^ patches)	×	×	6.5 (6 layers)	1.7
Fan et al. [16]	×	Fixed	×	64,000 patches	50	20	×	1.29
*Hybrid models*								
Wolterink et al. [72]	NVIDIA Titan X	Exponential	×	×	48	100	×	< 10
You et al. [83]	NVIDIA Titan Xp	Adaptive	×	100,100 patches	64	100	15 (2D model), 26 (3D model)	0.534 (2D model), 4.864 (3D model)
Du et al. [12]	NVIDIA Titan V	Fixed	×	4000 slices (140,000 patches)	32	20	×	0.08
Chi et al. [6]	NVIDIA Titan Xp, 12 GB	Time-based	×	4036 slices	×	×	×	1.84
Yin et al. [82]	NVIDIA RTX 2070	Fixed	×	2400 slices (11,648 patches)	32	100	14	×

### Patch extraction

In LDCT restoration, patches can better represent the local features of the image. Also, these patches will affect the denoising performance. In addition to that, patches boost the number of samples via the training data [45]. Therefore, generating overlapped patches is encouraged in most of the reviewed applications [3, 4]. Patches accelerate the convergence of the learning model dramatically due to the ability to make full use of limited CT data [23]. Tables 1 and 3 emphasize the patch sizes used in various LDCT restoration applications.

### Transfer learning

Transfer learning is a machine learning technique used to improve learning in a new learning model via the transmission of knowledge from another similar already learned model. Transfer learning can dramatically reduce the training time and avoid over-fitting the LDCT restoration model [30]. This study has revealed various transfer learning approaches implemented in various LDCT restoration applications. Among them, using a pre-trained network for transferring knowledge has been reported in several studies [6, 12, 20, 30, 77]. The VGG-19 [63] of ImageNet [9] has been used as the pre-trained network in those studies. However, the features generated by the VGG-based transfer learning approaches may not be relevant to the CT features. The main reason for that is those models were trained using natural images. Other than using a pre-trained model, Zhong et al. [91] and Shan et al. [58] have used a self-supervised learning model as a transfer learning strategy. In this approach, they have trained a CNN model using natural images with Gaussian noise. However, it can be concluded that using a self-supervised learning model to fine-tune the target model overcomes the drawback of using VGG-based pre-trained models.

### Batch normalization

Batch normalization is another technique used in LDCT restoration. It is used to improve training efficiency by reducing the statistical difference between the CT images [81]. Also, batch normalization contributes to faster convergence and reduce sensitivity to initiate the learning model [50]. Its ability to solve the internal covariate shift boosts the fast network convergence.

## Future research directions

Performance is an ever-growing requirement in LDCT restoration. In this regard, several knowledge gaps exist to address within the current LDCT restoration domain. First, the article explains the main issue that exists in supervised DL methods. Usually, NDCT data are used as the labeled data in supervised DL methods which are not free from noise and artifacts. Therefore, the denoising accuracy of most of the current supervised learning-based LDCT restoration algorithms is reduced by these retaining noise components in NDCT images. However, the application of migration learning can be declared as a potential technique to be experimented for restoring the noise and artifacts in NDCT images [28].

Proposing novel methods for training the DL models in an unsupervised manner is also considered as an open area in LDCT restoration. Alternatively, this will address the absence of paired data in the clinical setup. The literature emphasizes proposing the cyclic-GAN models and the definition of denoising-prior images from the NDCT as currently proposed solutions [35, 53, 67]. However, the efficiency and effectiveness of the defined denoising-priors depend on the quality of the training dataset. Moreover, a low-quality training dataset leads to generate fake or structure fragile CT results [53]. Thus, selecting a suitable dataset for defining denoising-priors is challenging and empirical [46]. Also, it is worth exploring the features shared between LDCT and NDCT images, such as sharpening and sparse information, when declaring denoising-priors to enhance the functionality of LDCT restoration.

Attention networks are a DL method for improving the performance of LDCT restoration, which got popular recently. Although the current attention-based DL methods have gained an acceptable visual performance in CT restoration, the quantitative results of those proposed methods are not optimal in some cases when comparing them based on PSNR and SSIM measurements. The main reason for that is the lack of attention given to the structural feature preservation and tolerate the pixel-wise loss functions during the model training [12, 42]. Therefore, the noise and structure deformation still appeared as the degradations in the restored CTs. Hence, proposing a multiple enhancement features attention-based DL models is significant as future research attempts to overcome this issue.

Generalizability directly affects improving the adaptability and clinical usability of the denoising application. Generally, it emphasizes how the proposed model can adapt to unseen data extracted from various generalizability levels, including different anatomies, noise levels, dimensions (2D, 3D or multi-dimensional), noise distributions, and vendors’ devices. The LDCT restoration applications reviewed in this study have been widely tested for different noise levels, image formats, and multi-anatomies. Hence, improving the generalizability of DL-based LDCT restoration algorithms for multiple scanners, organs, and imaging protocols are essential. Apart from that, exploring the ways to the reduction of metal artifacts and motion artifacts during the restoration is an open-ended question [28, 53].

Overall, it can be stated that the DL-based denoising techniques have provided benchmarked adaptive denoising solutions with a high visual performance. However, the hyper-parameters in DL networks such as the number of layers, number of filters, and different DL architectures are critical factors that affect the accuracy of the results. Therefore, it is essential to find a mechanism to initialize these hyper-parameters optimally to enhance the accuracy of LDCT restoration results. Also, the experiment on exploring the DL models with optimal hyper-parameters is an open research area [4].

In the context of medical imaging, the performance gained through transfer learning using the natural image-based pre-trained network is not optimal. The main reason for this is, the medical images are usually represented as texture-rich low-contrast images than natural images. However, it is recommended that targeted networks be trained with pre-trained task-specific networks to obtain optimal results [42]. In this approach, the target network can be trained with task-relevant similar images [12, 83]. However, developing a task-specific pre-trained network is challenging due to the difficulty of extracting large amounts of annotated medical image data. In addition to that, to improve the performance of the target network, cross-model transfer learning networks can also be recommended as a plausible solution. Finding the models for both task-specific and cross-model transfer learning has been still existed an open issue to address. Unlike the conventional cross-domain transfer learning models, task-specific or cross-modal transfer learning models will be able to match the exact features of the same domain, thereby improving the performance and accuracy of the denoising process.

## Conclusion

Noise and artifacts are one of the inevitable degradation factors in CT imaging. It reduces the visual quality of CT images by obstructing the accuracy of clinical judgments. DL-based LDCT restoration provides promising solutions to overcome this issue. Therefore, this study has presented a comprehensive review of DL-based LDCT restoration by focusing on several important themes. Initially, this review provided an overview of degradations in LDCT images. Then, it has emphasized the various DL techniques and architectures used in recent applications for LDCT restoration. Moreover, this study has presented sound comparisons of performance and functional aspects of DL-based LDCT restoration applications. Analysis results have shown that the GAN-based applications outperform the other DL-based LDCT restoration algorithms due to their multi-objective functions, flexibility to upgrade the generator architectures, and the multi-scale discriminator. Finally, this study has emphasized the open research problems and future research directions for prospective researchers to come up with new CT restoration-based research proposals that can improve computer-aided diagnostic accuracy.

## Glossary

CT: Computed Tomography
LDCT: Low-dose CT
SNR: Signal to Noise Ratio
NDCT: Normal-dose CT
DL: Deep Learning
MPGD: Mixed Poisson Gaussian distribution
BM3D: Block Matching Three Dimension
ML: Machine Learning
CNN: Convolutional Neural Networks
ReLU: Rectified Linear Unit
SCN: Stacked Competitive Network
ResNet: Residual Network
DenseNet: Dense Network
SSDA: Stacked Sparse Denoising Autoencoder
GAN: Generative Adversarial Network
SSIM: Structural Similarity Index Matrix
PSNR: Peak Signal to Noise Ratio
MSSIM: Mean Structural Similarity Index
